# Notes and News

**Published:** 1894-03-24

**Authors:** 


### Notes and News.

Collected and Communicated.

A meeting in opposition to the proposed Isolation Hospital for Richmond, Isleworth, and Heston, to be placed at Twickenham, was held recently. A petition has been drawn up, and one clause in it stands that "The proposal of two places in different counties to combine is an infringement upon the principles of county government and local self-government." The speakers at the meeting were strongly of the opinion that great injury would result to Twickenham by the establishment of a fever hospital in the vicinity, and a further attempt to discredit the choice of this site was made by a statement that the land was possessed by a promoter of the scheme, and that a far too high price was asked for it. The Local Government Board are about to meet and hold inquiry in the matter at Twickenham.

The scheme for a hydropathic hospital for London is again being aired in the columns of the *Echo*. Our advocate is most sanguine of the results which would accrue from the establishment of such an institution. He further appeals to all anti-vivisectionists to help, as he says, "the best way to damage the cause of vivisection is to prove that it is useless." Speaking of the treatment on the "hygienic system," as the writer calls it, for consumption, he points out that one year of experience of such a method carried out in some establishment would do more to "open the eyes of the public to what is possible in the treatment of consumption than twenty years talk." And "this treatment would then be adopted in all other hospitals and convalescent homes, and we should be able to place the treatment of consumption on a firm, scientific basis, capable of universal application." There is nothing to prevent the establishment of a hydropathic hospital in London, any more than any other special hospital, and if there is so crying a need for it, doubtless funds will be forthcoming. Whether Mr. Lovell, the writer we have quoted, will attract an intelligent class of supporters remains to be seen.

The last fortnightly meeting of the Metropolitan Asylums Board reported a decrease both as to fever and small-pox patients in the metropolis. In the case of fevers the decrease was 123; in the case of small-pox it amounted to five.

A list of such manufactures as are regarded as dangerous and insanitary is kept by the State in France. This classified list has been recently added to. Amongst the manufactures ranged in the first class is that of artificial silk.

An ambulance school was opened at the International Hospital, Paris, last week. This is the third school of the kind that has been established in that city. The second was inaugurated in connection with the Polytechnic of Paris. The schools are indebted to the liberality of the Municipal Council. These so-called ambulance schools are in reality regarded as nurse training schools for men and women both. The ceremony held last week was presided over by Mons. Albert Pétrot, Deputy for Paris.

An inquiry was held at Ealing last week on the proposed scheme to place a fever hospital for Chiswick and Gunnersbury, at Ealing. The site is undoubtedly approached by a very narrow lane. Not far off runs the public footpath to Harrow, and, further, it is said to be damp and occasionally flooded land. Ealing and Hanwell are not unlike other neighbourhoods in pleading a likely decrease in the value of house rent and building property, and they also put forward the more novel injury to dairy farming as likely to accrue.

The forty-first half-yearly report just issued of Dr. William Collingridge, Medical Officer of Health for the Port of London, shows that the continued existence of cholera on the Continent, rendered the half-year an extremely trying one. As a result of the inspection, during the six months twelve vessels were found to be infected with cholera, and dealt with accordingly. Of these five had actual cases on board at the time, and two were suspicious. Another medical man was stationed at Sheerness to inspect vessels entering the Medway, and had the cholera appeared in Holland, all passengers from thence to Queensborough would have been subjected to examination. During the outbreak at Hull, Grimsby, and Goole, special arrangements were made for visiting arrivals, as these vessels were not subject to a visit from the Customs.

During the six months thirty-six patients suffering from various infectious diseases had been removed from shipping to the Port Sanitary Hospital. The charge for maintenance of patients in this hospital has now been abolished. The hospital is provided with a steam disinfector of the Washington Lyon type, and in this are treated not only effects of patients, but the effects of those who have died or suffered from infectious disease either abroad or on the voyage. The disinfection of rags packed in hydraulically compressed bales and imported as merchandise was discontinued in August by order of the Local Government Board, there being no authenticated evidence of the transmission of cholera by means of these goods, so that whereas 2,188 bales were disinfected during the first six weeks of the half-year, only 442 have been so treated since August 11th. Numerous seizures of unsound food of various sorts were made, the goods being in all cases destroyed.

